# Effects on alcohol consumption of announcing and implementing revised UK low-risk drinking guidelines: findings from an interrupted time series analysis

**DOI:** 10.1136/jech-2020-213820

**Published:** 2020-11-01

**Authors:** John Holmes, Emma Beard, Jamie Brown, Alan Brennan, Petra S Meier, Susan Michie, Abigail K Stevely, Laura Webster, Penny F Buykx

**Affiliations:** 1 ScHARR, University of Sheffield, Sheffield, UK; 2 Department of Clinical, Educational and Health Psychology, University College London, London, UK; 3 Cancer Research UK Health Behaviour Research center, University College London, London, UK; 4 School of Humanities and Social Science, University of Newcastle Medical Society, Newcastle, Australia

**Keywords:** Alcohol, health promotion, outcome research evaluation, time-series, public health policy

## Abstract

**Background:**

In January 2016, the UK announced and began implementing revised guidelines for low-risk drinking of 14 units (112 g) per week for men and women. This was a reduction from the previous guidelines for men of 3–4 units (24–32 g) per day. There was no large-scale promotion of the revised guidelines beyond the initial media announcement. This paper evaluates the effect of announcing the revised guidelines on alcohol consumption among adults in England.

**Methods:**

Data come from a monthly repeat cross-sectional survey of approximately 1700 adults living in private households in England collected between March 2014 and October 2017. The primary outcomes are change in level and time trend of participants’ Alcohol Use Disorders Identification Test—Consumption (AUDIT-C) scores.

**Results:**

In December 2015, the modelled average AUDIT-C score was 2.719 out of 12 and was decreasing by 0.003 each month. After January 2016, AUDIT-C scores increased immediately but non-significantly to 2.720 (β=0.001, CI −0.079 to 0.099) and the trend changed significantly such that scores subsequently increased by 0.005 each month (β=0.008, CI 0.001 to 0.015), equivalent to 0.5% of the population increasing their AUDIT-C score by 1 point each month. Secondary analyses indicated the change in trend began 7 months before the guideline announcement and that AUDIT-C scores reduced significantly but temporarily for 4 months after the announcement (β=−0.087, CI −0.167 to 0.007).

**Conclusions:**

Announcing new UK drinking guidelines did not lead to a substantial or sustained reduction in drinking or a downturn in the long-term trend in alcohol consumption, but there was evidence of a temporary reduction in consumption.

## INTRODUCTION

The UK’s Chief Medical Officers updated their guidelines for low-risk alcohol consumption in 2016,^1^ following a review that was a key component of the Government’s 2012 alcohol strategy.^2^ The new guidelines for men and women recommend that ‘to keep health risks from alcohol to a low level, it is safest not to drink more than 14 units a week on a regular basis’ (Department of Health, p4).^1^ One UK unit is 10 mL or 8 g of pure ethanol and 14 units is approximately 6 pints of average strength beer, a bottle and a half of wine or 14 single measures of spirits. The new guidelines also recommend limiting the amount consumed on any single occasion, having several drink-free days each week and spreading consumption over 3 or more days if drinking as much as 14 units a week.^1^

The previous guidelines recommended that men should not regularly drink more than 3–4 units per day and women not more than 2–3 units per day.^3^ Therefore, the guidelines have changed in three main ways: by moving from a daily to a weekly guideline, by equalising the guideline for men and women and by reducing the guideline for men by approximately one-third.

Promotion of the revised guidelines has been limited. The UK Government announced and began implementing the guidelines in January 2016 via a press release that was titled ‘New alcohol guidelines show increased risk of cancer’ and which emphasised the risk of cancer for drinkers consuming only small amounts of alcohol.^4^ News and social media gave the announcement substantial attention, and most news coverage reported the guidelines in a factual manner.^5 6^ However, a minority of articles criticised the focus on cancer risks from moderate drinking and associated statements by the Chief Medical Officer for England, who suggested that drinkers must choose between wine and cancer.^5^ After the initial announcement, there was no mass media campaign or other large-scale activity to promote the guidelines by any organisation until two brief campaigns in September 2018 and March 2019, which are outside our study period. Alcohol producers also did not update the guidelines provided on product labels.^7 8^

This lack of promotional activity is not unusual as few countries promote their drinking guidelines through sustained campaigns. As a result, there is little agreement or evidence on the effectiveness of using guidelines as a public health intervention. Some public health stakeholders argue that drinking guidelines are ineffective in combatting pro-alcohol marketing, draw attention away from more effective interventions and are used by commercial actors to deliver ambiguous messages that may promote consumption.^9–11^ Others argue that guidelines provide people with important information about health risks, facilitate discussions within clinical practice and may change the debate around alcohol so as to make it easier to introduce effective alcohol control policies.^12 13^

Evidence to support either set of views is sparse and reviews note there have been no rigorous evaluations of the impact of producing, revising or promoting drinking guidelines on alcohol consumption or alcohol-related attitudes, norms or motivations.^,11 14^ The literature that does exist relies on relatively weak research designs that permit only limited causal inference, such as small numbers of annual cross-sectional surveys.^15–17^ Findings from such studies suggest that publishing drinking guidelines may improve public awareness and knowledge of the guidelines but does not affect alcohol consumption. Our own descriptive analyses of the short-term effects of the new UK guidelines up to January 2017 support this view and suggest that knowledge of the guidelines and preparedness to change behaviour increased initially, but these effects gradually diminished.^18 19^

The present study has two aims. First, to update to October 2017 our descriptive analysis of trends in drinkers’ awareness, knowledge and exposure to the guidelines and drinkers’ capability, opportunity and motivation to change their behaviour. Second, and the main focus, to use an interrupted time series analysis of 44 months of survey data to evaluate the longer-term impact of the new UK drinking guidelines on alcohol consumption.

## METHODS

Ethics approval for this research was granted by the University of Sheffield’s Research Ethics Committee (Ref: 006373)

### Data

This study uses data from the Alcohol Toolkit Study (ATS), a monthly cross-sectional survey of ~1700 adults aged 16+ living in private households in England.^20^ The ATS selects respondents using a hybrid between random location sampling and quota sampling of households within locations. It has collected data since March 2014 and incorporated additional questions for the present study for 24 months from November 2015. In the absence of subsequent promotional activity, the intervention point for all analyses is the announcement of revised guidelines in January 2016. This means we have 22 months of preintervention data and 22 months of postintervention data for most measures (March 2014 to October 2017), but only 2 months of pre-intervention data for some guideline-specific questions described below (November 2015 to October 2017).

### Measures

The updated descriptive analyses use previously described ATS measures, included in the survey from November 2015, to examine drinkers’ awareness, knowledge and exposure relating to guidelines and their readiness to change behaviour.^18 19^ Briefly, the ATS measured awareness by asking whether drinkers have heard of the guidelines. Those that had were asked what they think the guideline consumption level is, with either 14 units per week or 2 units per day classed as correct. All drinkers who stated a guideline consumption level, whether correct or incorrect, were then asked in which of 11 locations they had seen this guideline in the last month (see table 1 for locations). All drinkers were also asked 10 questions designed to assess their capability, opportunity and motivation to change their drinking, in line with the Capability Opportunity Motivation - Behaviour (COM-B) model of behaviour change (see online supplementary table A1 for details).^21^

10.1136/jech-2020-213820.supp1 Supplementary data

The primary outcome for the evaluation analysis is alcohol consumption measured by participants’ Alcohol Use Disorders Identification Test—Consumption (AUDIT-C) scores. AUDIT-C is a validated screening test for heavy drinking and/or active alcohol abuse or dependence, which has been shown to respond to short-term changes in respondents’ drinking.^22^ The test asks three questions that address drinkers’ alcohol consumption frequency, their quantity of alcohol consumed on a typical drinking day and their frequency of heavy drinking. Responses to each question are scored from 0 to 4 and then summed to give the total AUDIT-C score, ranging from 0 to 12.

**Table 1:** Preintervention and postintervention values for measures and significance of preintervention and postintervention differences

Measure	N*	Preintervention*	Postintervention*	χ² or t-statistic	P value
Total N (March 2014 to October 2017)	74 388	36 905	37 483		
Total N (November 2015 to October 2017)	40 832	3349	37 483		
Demographics (March 2014 to October 2017)	74 388				
Men		49.0	49.0		
Women		51.0	51.0	7.9	**<0.01**
16–34 years old		31.1	31.1		
35–64 years old		48.1	47.9		
65+ years old		20.8	21.1	105.0	**<0.01**
Drinker in last six months (November 2015 to October 2017)	26 335	**68.1**	**65.7**	**6.5**†	**0.01**
Aware of guidelines	26 306	86.0	87.0	1.6	0.20
Identifies guidelines as 14 units per week	22 466	**21.4**	**23.9**	**16.6**	**<0.01**
Last month exposure to guideline via:	19 548				
Product label		20.1	22.1	2.6	0.11
TV or radio		**34.2**	**39.1**	**11.8**	**<0.01**
Newspapers or magazines		15.8	17.6	2.5	0.11
Websites or social media		6.0	7.5	3.7	0.05
Shops or supermarkets		8.3	8.3	0.0	0.98
Pubs, bars or nightclubs		**13.0**	**11.1**	**4.5**	**0.03**
Place of work or study		6.5	7.4	1.1	0.30
Health professional		8.9	8.0	1.5	0.22
Health service		11.1	11.7	0.5	0.47
Friends, family or colleagues		6.4	7.5	2.0	0.16
Other		**1.4**	**0.6**	**9.4**	**<0.01**
In any location		**71.7**	**76.5**	**20.9**	**<0.01**
COM-B measures					
Can drink only up to two units before harm	24 245	34.2	35.6	1.26	0.26
Easy to drink moderately	26 203	82.5	82.0	0.24	0.63
Tracks units	26 274	26.7	25.9	0.46	0.50
Lifestyle makes it easy	26 143	**76.9**	**79.4**	**6.5**	**0.01**
Know where to get advice	26 233	**78.6**	**82.3**	**16.0**	**<0.01**
Trying not to drink more than is good for me	26 063	**48.7**	**51.2**	**3.8**	**0.05**
Intends to drink moderately	26 249	77.0	77.5	0.2	0.65
Wants to avoid drinking excessively	26 247	76.0	76.4	0.2	0.69
Trying to avoid drinking excessively	26 224	39.3	39.6	0.1	0.77
Concerned by drinking too much	26 256	24.4	23.8	0.3	0.56
Drinker in last 6 months (March 2014 to October 2017)	48 875	**70.4**	**65.7**	**163.7**	**<0.01**
AUDIT-C score (mean)	48 696	**4.1**	**4.4**	**−10.2**†	**<0.01**
Litres ethanol per adult 16+ (mean)		0.81	0.79	0.64†	0.26
Assaults (mean)		2261.7	2197.6	1.0†	0.16
Alcohol poisonings (mean)		190.9	186.2	0.7†	0.25
November 2015 to October 2017					
Graduated frequency weekly units (mean)	22 404	9.7	9.9†	−0.3	0.61
Exceeding 14 units per week	22 404	19.5	21.0	2.2	0.19

*Unweighted Ns and weighted column percentages unless identified as means.

†Test statistic is the t-statistic. Rows in bold are significant preintervention/postintervention differences at the 0.05 level.

To test the robustness of our evaluation results, we examine four secondary outcomes: (1) average weekly alcohol consumption in units measured using graduated frequency questions included in the ATS between November 2015 and October 2017 (see online supplementary table A2 for details); (2) monthly litres of pure alcohol released for sale per adult aged 16+, as derived from UK alcohol taxation records; (3 & 4) monthly number of hospitalisations in England for alcohol poisoning International Classification of Diseases (ICD)-10: T51.0, T51.1, T51.9) and assaults (ICD-10: X85-Y09), calculated from National Health Service (NHS) Digital’s Hospital Episode Statistics. Taxation and hospitalisation data are from March 2014 to October 2017.

Adjusted evaluation models control for monthly retail price indices provided by the Office for National Statistics for four beverage categories: on-trade beer, off-trade beer, on-trade wine and spirits, and off-trade wine and spirits. They also control for weather using the Met Office’s Hadley Centre Central England Temperature, which records the average monthly temperature across an area of Central England approximately enclosed between London, Bristol and Lancashire.

### Analysis

The updated descriptive analyses plot the monthly trend in each measure and then use ᵡ² and t-tests to assess differences between the pre-intervention and post-intervention periods. All descriptive analyses used weighted data.

The primary evaluation analysis uses the individual-level survey data within an interrupted time series design to assess the effect of the January 2016 announcement and implementation of revised drinking guidelines on AUDIT-C scores in the whole population. We analyse the data using generalised additive models (GAM; R package *mgvc*). Each GAM models the trend in AUDIT-C scores in the preintervention period, any immediate step change in AUDIT-C scores after the guideline announcement and any change in the trend in AUDIT-C scores in the postintervention period relative to the preintervention period. We run models adjusted for seasonality only and then models adjusted for the monthly price indices, for temperature and for both.

The analyses of secondary outcome measures use the same approach with some adjustments. As there are only 2 months of preintervention graduated frequency data, we cannot model the preintervention trend. Instead, we conduct a simple pre/postintervetion analysis using GAMs and adjust for covariates as above. For the taxation and hospitalisation data, we run aggregate-level models. Autocorrelation was present for all three outcomes and the final models account for this by extending the GAM to a Generalised Additive Mixed Model.

Additional preplanned sensitivity analyses test the robustness of our results. First, we test whether any change in the AUDIT-C trend precedes, coincides with or follows the guideline announcement by using the *segmented* package in R to identify statistically any significant breakpoints in the AUDIT-C trend, rather than assuming a breakpoint in January 2016. We then re-run the primary analysis using the newly identified breakpoint. Second, we test for a pulse effect (ie, an immediate but temporary change) following the guideline announcement by running GAMs with pulses lasting 2 and 3 months and assuming a constant underlying time trend. Third, we run segmented polynomial GAMs to test whether a quadratic or cubic model fits the data better than the linear model used in the primary analysis.

We also conduct three unplanned sensitivity analyses. First, we re-ran the primary analysis for men and women separately as the guidelines were only changed substantially for men. Second, there is greater variability in postintervention AUDIT-C scores than anticipated and so we test whether using postintervention data up to February 2018 affects the results. Third, we test when the identified pulse effects dissipate.

## RESULTS

Table 1 provides numbers of respondents, preintervention and post intervention values, and significance tests of differences for all measures used in the descriptive and evaluation analyses. The findings are discussed in detail below.

### Updated descriptive analyses

**Figure 1:**
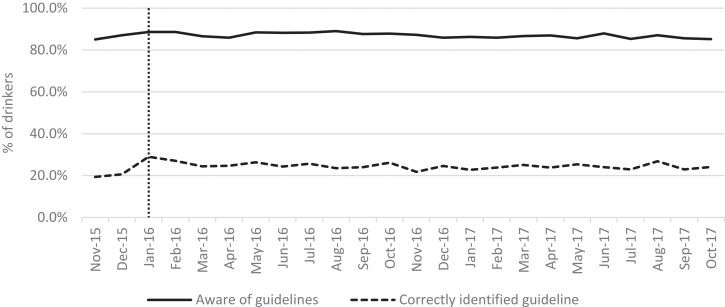
Monthly trend in awareness and knowledge of drinking guidelines among drinkers.

**Figure 2:**
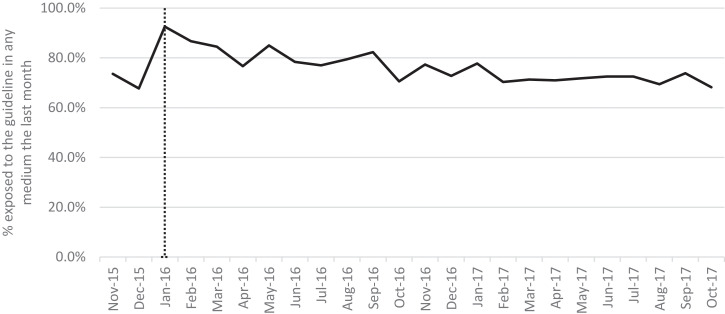
Past month exposure to guidelines among drinkers able to state a guideline.

Awareness of the existence of guidelines was high at over 85% across the study period but did not change significantly postintervention (figure 1, table 1). The proportion of drinkers who correctly identified the guideline increased significantly from 21.4% preintervention to 23.9% postintervention, but remained below 25% throughout the study period. After the announcement, 76.5% of those who answered the exposure question reported seeing the guideline in at least one place in the last month, but exposure peaked in January 2016 and returned to preintervention levels by October 2017 (figure 2). Exposure was most common via TV or radio and less than a quarter of respondents reported last-month exposure via any other medium in either the preintervention or postintervention period.

There were few significant changes in measures of capability, opportunity and motivation between the preintervention and postintervention periods (table 1). The proportion of drinkers who said their lifestyle makes it easy to drink three or fewer units a day, who knew where to get advice on how to cut down on drinking and who were trying not to drink more than is good for them all increased significantly, but by less than four percentage points. Time trend graphs for all measures are available in online supplemental figures A1–A5.

### Analysis of primary outcome

**Figure 3:**
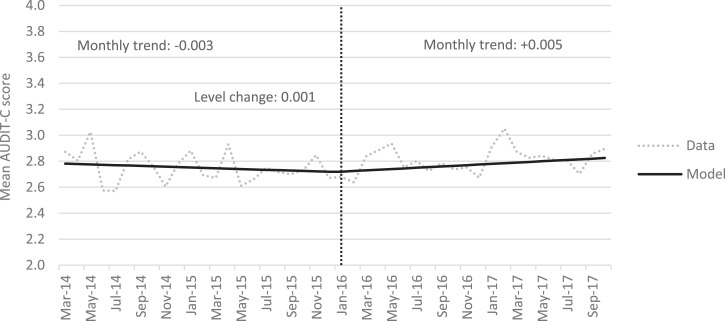
Alcohol Use Disorders Identification Test—Consumption (AUDIT-C) scores before and after the announcement of revised drinking guidelines.

The trend in AUDIT-C scores was largely stable across the study period, with an average score of 2.752 before January 2016 and 2.809 afterwards (figure 3, table 1). The analysis tested for a step change in AUDIT-C scores immediately after the announcement and found no significant change (table 2). It also tested for a change in the trend in AUDIT-C scores and found a significant effect: the preintervention trend was for a decrease in AUDIT-C scores of 0.003 per month whereas the postintervention trend was for an increase of 0.005. This is equivalent to an additional 0.5% of the population increasing their AUDIT-C score by 1 point each month by, for example, drinking 4+ times per week rather than 2–3 times per week. The results did not change after adjusting for temperature but the change in trend was not significant when controlling for alcohol prices or in the fully adjusted model.

**Table 2:** Results of interrupted time series analyses for primary (AUDIT-C scores) and secondary outcomes

	Unadjusted				Adjusted for temperature				Adjusted for off and on prices of beer and wine/spirits				Adjusted for temperature and on and off prices of beer and wine/spirits			
	β	Lower CI	Upper CI	P value	β	Lower CI	Upper CI	P value	β	Lower CI	Upper CI	P value	β	Lower CI	Upper CI	P value
*AUDIT-C scores*																
Intercept	2.785	2.723	2.847	<0.001	2.740	2.554	2.926	<0.001	5.055	−5.893	16.003	0.365	3.484	−7.775	14.743	0.544
Preintervention trend	−0.003	−0.008	0.002	0.306	−0.002	−0.007	0.003	0.325	<0.001	−0.010	0.010	0.975	<0.001	−0.010	0.010	0.977
Step-level change	0.001	−0.079	0.099	0.824	0.011	−0.078	0.100	0.805	0.027	−0.081	0.135	0.619	0.029	−0.079	0.137	0.596
Change in trend	0.008	0.001	0.015	0.015	0.008	0.001	0.015	0.020	0.007	−0.008	0.022	0.343	0.008	−0.007	0.023	0.314
*Graduated frequency*																
Intercept	9.861	9.134	10.588	<0.001	9.737	8.882	10.592	<0.001	−0.567	−58.095	56.961	0.985	7.858	−62.354	78.070	0.826
Step-level change	0.105	−0.652	0.862	0.785	0.089	−0.671	0.849	0.818	0.047	−0.715	0.809	0.905	−0.007	−0.799	0.785	0.987
*Alcohol taxation data*																
Intercept	0.797	0.776	0.818	<0.001	0.77	0.679	0.861	<0.001	7.696	−1.32	16.713	0.105	7.364	−2.242	16.97	0.144
Preinter trend	−0.001	−0.002	0.001	0.405	−0.001	−0.002	0.001	0.451	−0.007	−0.015	0.001	0.116	−0.006	−0.015	0.003	0.174
Step-level change	0.006	−0.025	0.036	0.715	0.005	−0.026	0.037	0.747	−0.060	−0.144	0.024	0.174	−0.056	−0.144	0.032	0.223
Change in trend	0.001	−0.001	0.004	0.248	0.001	−0.001	0.004	0.294	0.003	−0.007	0.013	0.559	0.003	−0.008	0.014	0.577
*Assault hospitalisations*																
Intercept	2249	2160	2342	<0.001	1786	1592	2004	<0.001	488×10^4^	4395	543×10^7^	<0.001	236 874	739	759×10^5^	<0.001
Preinter trend	1.000	0.997	1.003	0.934	1.001	0.998	1.003	0.757	1.002	0.996	1.008	0.546	1.001	0.996	1.006	0.807
Step-level change	0.927	0.875	0.983	0.015	0.927	0.878	0.978	0.009	0.941	0.885	1.000	0.061	0.925	0.875	0.977	0.009
Change in trend	1.005	1.001	1.009	0.030	1.004	1.000	1.008	0.080	0.997	0.989	1.005	0.440	0.999	0.992	1.007	0.867
*Alcohol poisoning hospitalisations*																
Intercept	173	156	192	<0.001	149	129	172	<0.001	86 236	0	415×10^8^	0.098	6267	0	569×10^7^	0.220
Preinter trend	1.009	1.001	1.017	0.040	1.008	1.000	1.016	0.061	1.009	0.996	1.022	0.189	1.008	0.995	1.022	0.221
Step level change	0.846	0.735	0.975	0.026	0.874	0.760	1.004	0.064	0.862	0.749	0.993	0.047	0.868	0.751	1.004	0.064
Change in trend	0.996	0.984	1.007	0.443	0.995	0.983	1.006	0.383	0.992	0.973	1.011	0.413	0.993	0.974	1.013	0.500

Rows in bold are significant effects at the 0.05 level. All models control for seasonality.

AUDIT-C, Alcohol Use Disorders Identification Test—Consumption.

### Analysis of secondary outcomes

The analyses tested for a change between the preintervention and postintervention periods in units of alcohol consumed per week measured using graduated frequency questions and found no significant effect (table 2). They also found no significant step change or change in trend for the measure of alcohol released for sale per capita. For incidence of assault hospitalisation, there was a significant step change and change in trend: assault hospitalisations decreased by 7.3% immediately after the guideline announcement, but this was partly counteracted by a change from a flat preintervention trend to a 0.5% increase in the incidence of hospitalisations each month. Only the step change remained significant in the adjusted models. There was also a significant step change in the incidence of alcohol poisoning hospitalisations after the guideline announcement but there was no significant change in trend. The incidence of alcohol poisoning hospitalisations fell by 15.4% immediately after the announcement, but this change was no longer significant after adjusting for the temperature trend.

### Sensitivity analyses

We tested for an alternative breakpoint in the AUDIT-C score trend and identified that consumption started to increase from June 2015, 7 months before the guideline announcement. When re-conducting the primary analysis using this breakpoint, the AUDIT-C scores showed no significant trend until June 2015 and then increased by 0.011 units per month thereafter (online supplementary figure A6). We also tested for pulse effects at 2 and 3 months and found that AUDIT-C scores were significantly lower 2 and 3 months postintervention, but these pulse effects dissipated after 4 months (online supplementary figure A7). Other preplanned robustness checks confirmed the results of the primary analysis or, in the case of the gender-stratified primary analysis, found no significant effects. The online supplementary tables A3–A7 provides numerical results for all sensitivity analyses.

## DISCUSSION

These findings suggest that announcing and implementing revised UK drinking guidelines in January 2016 did not lead to substantial or sustained changes in alcohol consumption. There was, however, some evidence of a small short-term reduction in consumption lasting 4 months. The results also suggest there were no substantial or sustained changes in any other key measures, such as drinkers’ awareness of, knowledge of, or exposure to guidelines or their preparedness to change their drinking behaviour. Although the primary and secondary analyses did detect some significant changes in outcome measures, notably in alcohol-related hospital admissions, these were not confirmed by alternative outcome measures and were often not present after controlling for confounding or in robustness checks.

The strengths of this study include the use of a monthly time series of nationally representative survey data across a 44-month period, the use of multiple proximal and distal outcome measures and the use of robust statistical analyses that permit control for important confounders and detailed exploration of temporal processes, such as pulse effects and alternative breakpoints. There are also a number of limitations to the analysis. First, AUDIT-C scores and the graduated frequency measure are subject to self-report biases, which may interact with the guideline announcement if drinkers seek to present themselves as drinking within the guidelines. However, our results do not support this possibility and we also include an objective measure of alcohol consumption derived from taxation data. Second, the AUDIT-C questions also referred to drinking during the previous 6 months, which may dilute any short-term intervention effect but should not prevent detection of a sustained change in consumption. Third, January is typically a light drinking month while December is a heavy drinking month and this seasonality may confound model estimates. Our time series is relatively short and so our controls for seasonality may not adequately address this problem if seasonality varies substantially between years. This may explain the anomalous results for hospitalisations. Fourth, our results may not generalise to scenarios where health authorities actively promote drinking guidelines after announcing them or do not link the guidelines to cancer risks. However, sustained promotional activity is uncommon in most countries, while using health risks to communicate guidelines is commonplace. Therefore, we are likely to be evaluating a typical intervention scenario.

Although the lack of impact from the new guidelines may be attributed to their limited promotion, it also aligns with a wider literature on the ineffectiveness of providing alcohol-related health information. Studies of mass media campaigns, mandatory health warning labels for alcoholic products and school-based education programmes all suggest that such interventions have little impact on alcohol consumption.^11 14^ This contrasts with evidence from other areas of public health, including smoking, nutrition and physical activity, where some information-based approaches, particularly mass media campaigns and health warnings, have been effective in certain contexts.^14 23^ The research literature offers three broad explanations for this. First, that alcohol-related health promotion is undermined in an environment where pro-alcohol marketing is highly prevalent.^11^ Second, that alcohol-related health promotion is often poorly designed. For example, labels may be difficult to read or easy to ignore and messages may have little regard for drinkers’ understanding of key concepts such as units or standard drinks.^24 25^ Third, that drinkers reject alcohol-related health promotion because it aligns poorly with their drinking practices and motivations and gives little regard to positive or pleasurable aspects of drinking.^26 27^

These explanations suggest that, as was the case with information-based approaches to tobacco control,^23^ promoting drinking guidelines may only be an effective intervention within wider strategies that introduce effective alcohol control measures. Nonetheless, materials to promote drinking guidelines can be improved by increasing their visibility and making it easier for drinkers to understand, assimilate and use key concepts. Researchers have begun to test alternative labels and guideline-related message to achieve this but the evidence remains weak.^28^ Furthermore, there remains a need for research and reflection on drinkers’ responses to guidelines and whether, how and why they make use of them within contemporary drinking practices. More generally, further robust studies are required evaluating the impact of announcing or promoting drinking guidelines on alcohol consumption and influences on behaviour change.

## CONCLUSIONS

Announcing and implementing revised UK drinking guidelines did not lead to substantial or sustained changes in drinkers’ alcohol consumption. There were also no substantial and sustained changes in drinkers’ awareness and knowledge of the guidelines, their exposure to the guidelines or their preparedness to change their alcohol consumption.

**What is already known on this subject**

- Literature reviews consistently find that there have been no rigorous evaluations of the impact of producing, revising or promoting drinking guidelines on outcomes including alcohol consumption, alcohol-related attitudes, norms or motivations, or alcohol-related harm. The limited evidence available suggests that announcing or promoting drinking guidelines may lead to increased awareness and knowledge of guidelines, but not to changes in alcohol consumption. Higher quality evidence is needed as drinking guidelines are a major component of government alcohol strategies in many high-income countries.

**What this study adds**

- In a typical implementation scenario, where drinking guidelines are not actively promoted, announcing new guidelines did not lead to substantial or sustained changes in alcohol consumption. There were also no significant changes in other key outcomes such as drinkers’ awareness of, knowledge of or exposure to the guidelines. There was, however, some evidence of a small, short-term reduction in alcohol consumption lasting for 4 months after the guideline announcement.
