# Supplementary material for: Effects on alcohol consumption of announcing and implementing revised UK low-risk drinking guidelines: findings from an interrupted time series analysis
Source: J Epidemiol Community Health. 2020 Nov 1;74(11):942–9. doi: 10.1136/jech-2020-213820 (PMC7576577; doi:10.1136/jech-2020-213820)
Supplement: Supplementary data [file jech-2020-213820s001.pdf]

APPENDICES

Table A1: Questions and response options for measures of COM-B model of behaviour change.

COM-B dimension	Response options
**Capability**	
Knowledge	
Item 1: What do you think is the most number of units you can personally drink in a day on a regular basis before it does significant harm to your health?	1* – 7+ units<br>Don't know
Perceived capability	
Item 2: How easy or difficult do you personally find it to drink three or fewer units of alcohol a day?	1. Extremely difficult – 7. Extremely easy*
Skills	
Item 3: How often, if at all, do you keep track of how many units of alcohol you personally drink each week?	1. Never – 7. Always*
**Opportunity**	
Social opportunity	
Item 4: How easy or difficult do you think your lifestyle makes it for you to personally drink three or fewer units of alcohol a day?	1. Extremely difficult – 7. Extremely easy*
Item 5: Do you know where to go if you wanted advice or information on how to cut down on your drinking of alcoholic drinks?	1. I have no idea – 5. Yes, definitely*
**Motivation**	
Reflective motivation	
Item 7: To what extent are you actively trying to avoid drinking more alcohol than is good for you?	1. Not at all – 5. Definitely*
Item 9: To what extent do you intend to keep your drinking within safe limits?	1. Not at all – 5. Definitely*
Automatic motivation	
Item 6: To what extent do you want to avoid drinking more than is good for you rather than just thinking that you should?	1. Not at all – 5. Definitely*
Item 8: To what extent do you want to keep your drinking within safe limits?	1. Not at all – 5. Definitely*
Item 10: Nowadays how concerned, if at all, are you about drinking more units of alcohol than is good for you?	1. Not at all concerned – 5. Definitely concerned*

**Indicates end of scale considered positive when creating dichotomised measures.*

Table A2: Questions and response options for graduated frequency measure of mean weekly alcohol consumption.

Question		Response options
1.	On how many days, if any, did you personally drink a drink containing alcohol in the last four weeks?	0-28
2.	As you may be aware, the amount of alcohol in contained in a drink is measured in units. What was the maximum number of units you personally consumed on any one day when drinking an alcoholic drink or drinks in the last four weeks?<br><br>If you need to remind yourself of the definition of a 'standard drink' or 'unit', please see the definitions on the show prompt.	1-60
3.	You mentioned that in the last four weeks, you personally had a drink containing alcohol on X days. On how many days, if any, in the last four weeks did you personally drink:<br>- 51-60 units<br>- 41-50 units<br>- 31-40 units<br>- 21-30 units<br>- 16-20 units<br>- 11-15 units<br>- 8-10 units<br>- 5-7 units<br>- 3-4 units<br>- 1-2 units<br>- (Questions start at appropriate level give response to question #2)	0-28<br>Sum of all responses must equal response to #1.

Table A3: Results of the gender-stratified interrupted time series analyses for AUDIT-C scores.

	Unadjusted				Adjusted for temperature				Adjusted for off and on prices of beer and wine/spirits				Adjusted for temperature and on and off prices of beer and wine/spirits			
	β	Lower CI	Upper CI	*P value*	β	Lower CI	Upper CI	*P value*	β	Lower CI	Upper CI	*P value*	β	Lower CI	Upper CI	*P value*
AUDIT-C scores (men)																
Intercept	3.228	3.151	3.305	<0.001	3.351	3.181	3.521	<0.001	12.037	3.560	20.514	0.005	12.536	0.271	24.441	0.045
Pre-intervention trend	0.002	-0.004	0.008	0.609	-0.002	-0.009	0.005	0.573	0.008	-0.008	0.024	0.301	0.008	-0.008	0.024	0.308
Step level change	0.039	-0.096	0.174	0.571	0.028	-0.107	0.163	0.689	0.116	-0.052	0.284	0.175	0.117	-0.052	0.286	0.175
Change in trend	0.001	-0.001	0.003	0.200	0.009	-0.001	0.019	0.070	-0.004	-0.023	0.015	0.668	-0.004	-0.025	0.017	0.678
AUDIT-C scores (women)																
Intercept	2.235	2.172	2.298	<0.001	2.318	2.181	2.455	<0.001	2.456	-2.333	7.245	0.315	1.328	-3.903	6.559	0.619
Pre-inter trend	0.001	-0.004	0.006	0.697	-0.002	-0.008	0.004	0.606	<0.001	-0.010	0.010	0.966	0.002	-0.009	0.013	0.720
Step level change	0.011	-0.099	0.121	0.848	0.005	-0.105	0.115	0.932	0.019	-0.099	0.137	0.756	0.017	-0.104	0.138	0.789
Change in trend	<0.001	-0.002	0.002	0.579	0.006	-0.002	0.014	0.159	0.004	-0.009	0.017	0.563	0.005	-0.008	0.018	0.431

Note: Rows in bold are significant effects at the 0.05 level. All models control for seasonality.

Table A4: Results of the iterative segmented regression analysis assessing the impact of the new drinking guidelines on AUDIT-C scores at alternative breakpoints.

	**β**	**Lower CI**	**Upper CI**	***P value***
Intercept	2.806	2.739	2.873	<0.001
Trend up to June 2015	-0.005	-0.011	0.001	0.059
**Trend after June 2015**	**0.011**	**0.003**	**0.019**	**0.006**

Table A5: Results of the pulse regression analysis assessing the impact of the new drinking guidelines on AUDIT-C scores.

	**β**	**Lower CI**	**Upper CI**	***P value***
Intercept	2.740	2.697	2.783	<0.001
**Underlying trend**	**0.002**	**<0.001**	**0.004**	**0.009**
**Pulse effect 2 months**	**-0.184**	**-0.292**	**-0.076**	**0.001**
**Pulse effect 3 months**	**-0.126**	**-0.218**	**-0.034**	**0.007**

Table A6: Results of the segmented regression analysis assessing the impact of the new drinking guidelines on AUDIT-C scores with an extended post-intervention period.

	**β**	**Lower CI**	**Upper CI**	***P value***
Intercept	2.785	2.722	2.848	<0.001
Pre-intervention trend	-0.002	-0.007	0.003	0.342
Step level change	-0.015	-0.099	0.069	0.731
**Change in trend**	**0.001**	**0.004**	**0.016**	**0.001**

Table A7: Results of the pulse regression analysis assessing the impact of the new drinking guidelines on AUDIT-C scores

	**β**	**Lower CI**	**Upper CI**	***P value***
Intercept	2.740	2.697	2.783	<0.001
**Underlying trend**	**0.002**	**<0.001**	**0.004**	**0.009**
**Pulse effect 2 months**	**-0.184**	**-0.292**	**-0.076**	**0.001**
**Pulse effect 3 months**	**-0.126**	**-0.218**	**-0.034**	**0.007**
**Pulse effect 4 months**	**-0.087**	**-0.167**	**-0.007**	**0.032**
Pulse effect 5 months	-0.042	-0.114	0.030	0.246
Pulse effect 6 months	-0.035	-0.101	0.031	0.290

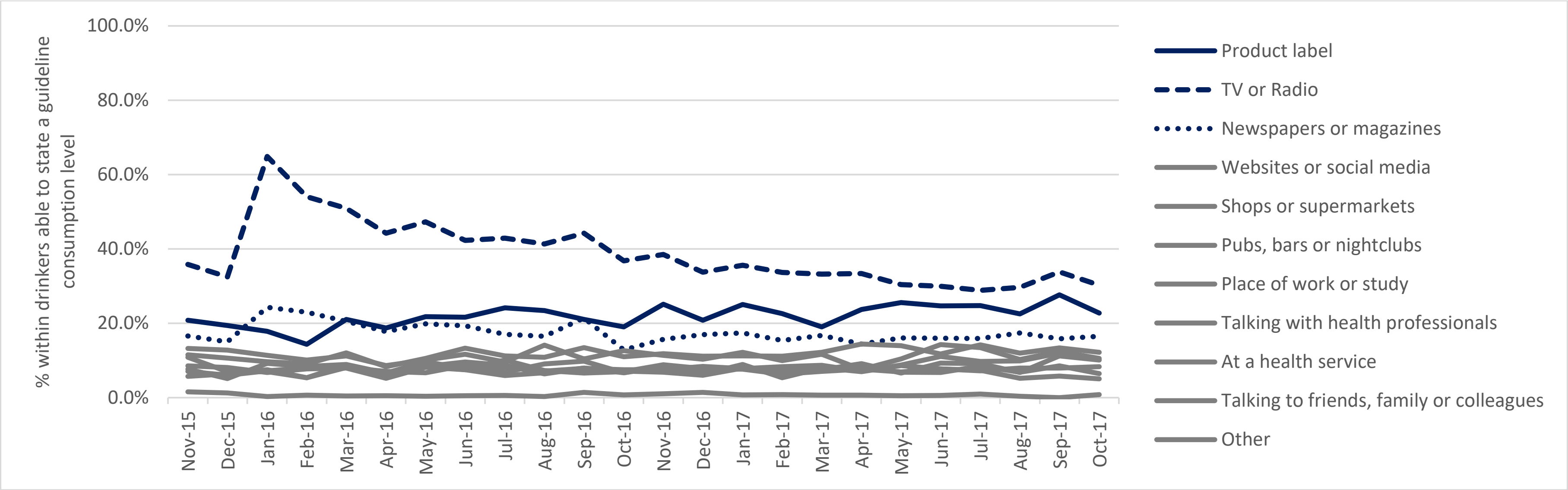

% within drinkers able to state a guideline consumption level
100.0%
80.0%
60.0%
40.0%
20.0%
0.0%
Nov-15
Dec-15
Jan-16
Feb-16
Mar-16
Apr-16
May-16
Jun-16
Jul-16
Aug-16
Sep-16
Oct-16
Nov-16
Dec-16
Jan-17
Feb-17
Mar-17
Apr-17
May-17
Jun-17
Jul-17
Aug-17
Sep-17
Oct-17
Product label
TV or Radio
Newspapers or magazines
Websites or social media
Shops or supermarkets
Pubs, bars or nightclubs
Place of work or study
Talking with health professionals
At a health service
Talking to friends, family or colleagues
Other

Figure A1: Trend in exposure to drinking guidelines over the past month in different places among drinkers able to state a guideline consumption level (correctly or incorrectly) within the Alcohol Toolkit Study.

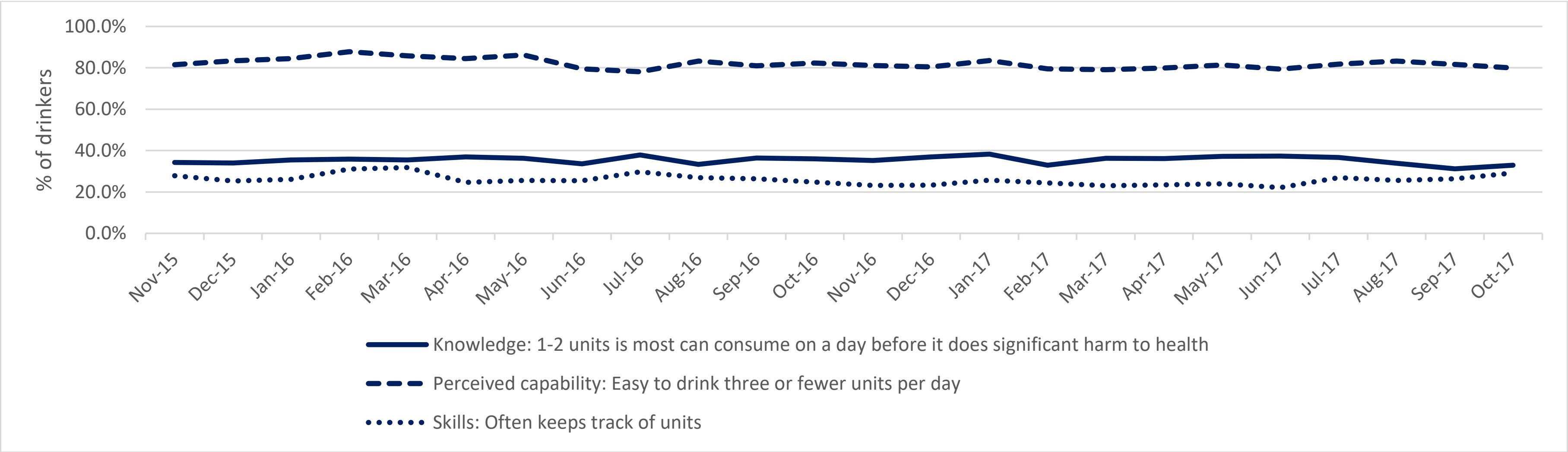

Figure A2: Monthly trend in COM-B capability measures among drinkers in the Alcohol Toolkit Study.

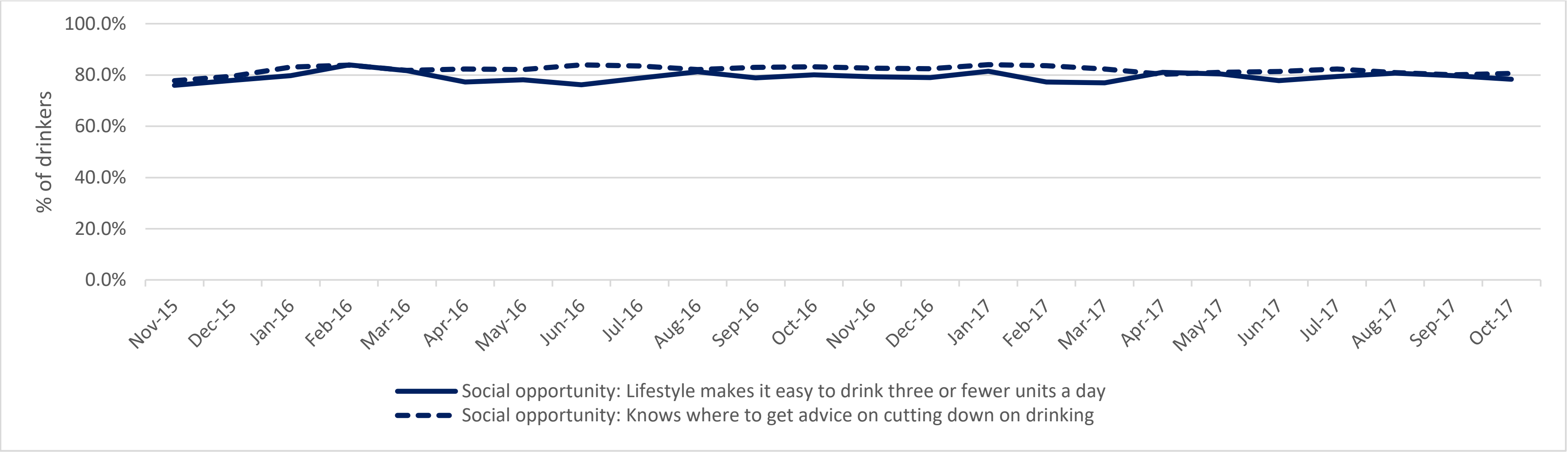

Figure A3: Monthly trend in COM-B opportunity measures among drinkers in the Alcohol Toolkit Study

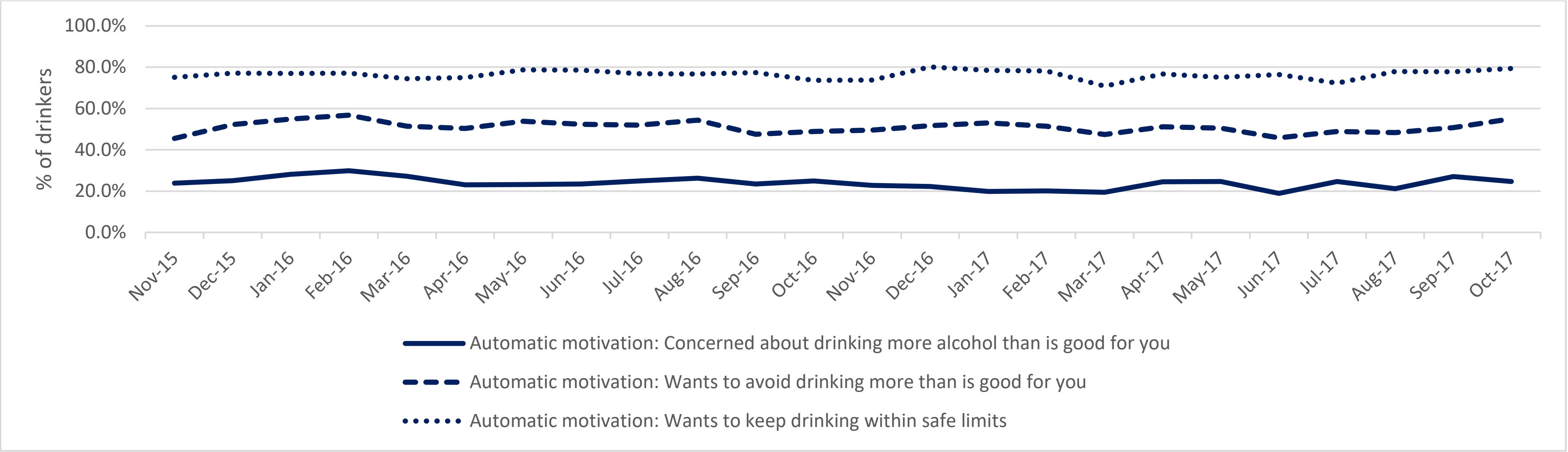

Figure A4: Monthly trend in COM-B automatic motivation measures among drinkers in the Alcohol Toolkit Study.

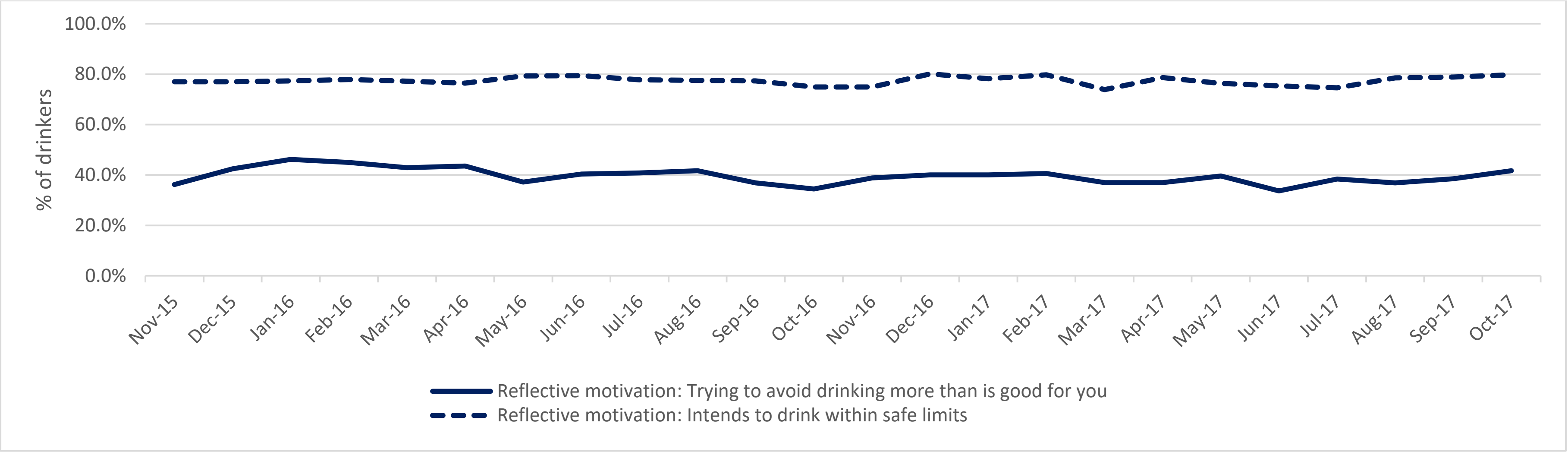

Figure A5: Monthly trend in COM-B reflective motivation measures among drinkers in the Alcohol Toolkit Study.

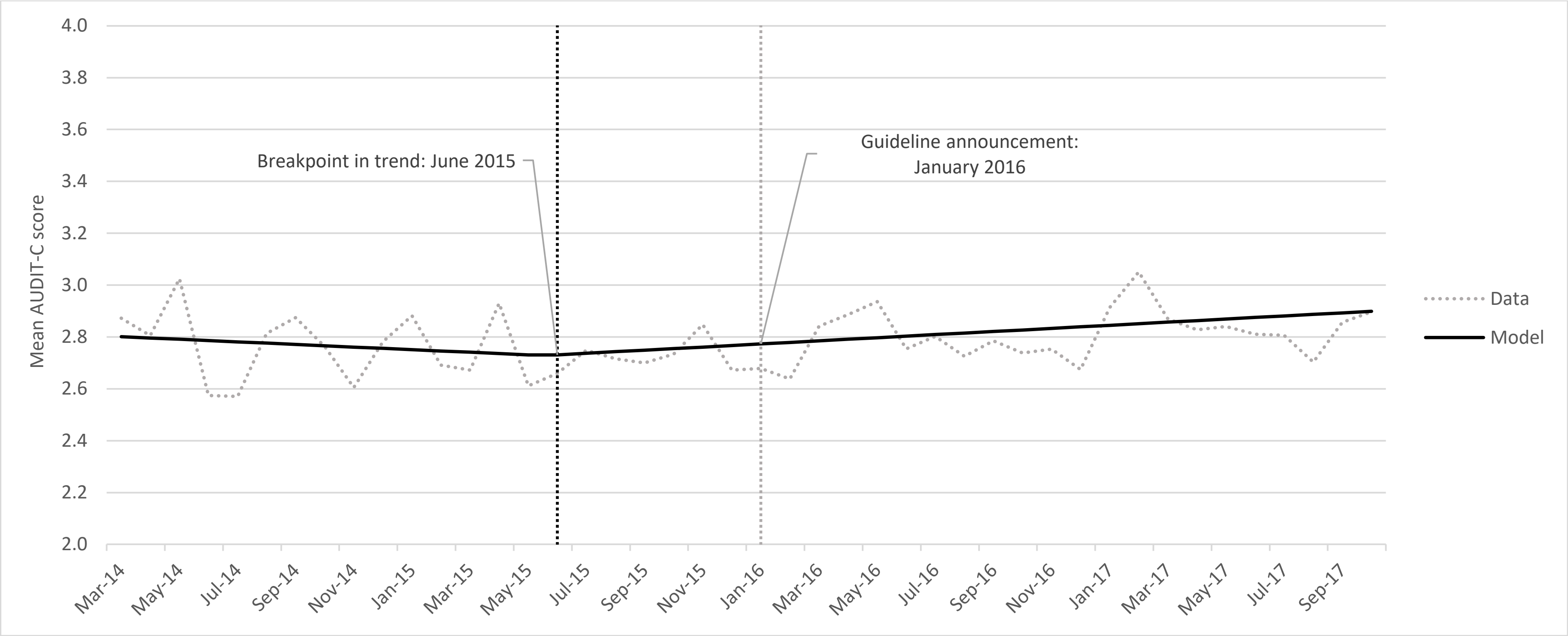

Figure A6: Secondary analysis showing statistically identified alternative breakpoint in the trend AUDIT-C scores in June 2015.[1]

[1] Model line shows the trend derived from model parameters after controls for seasonality.

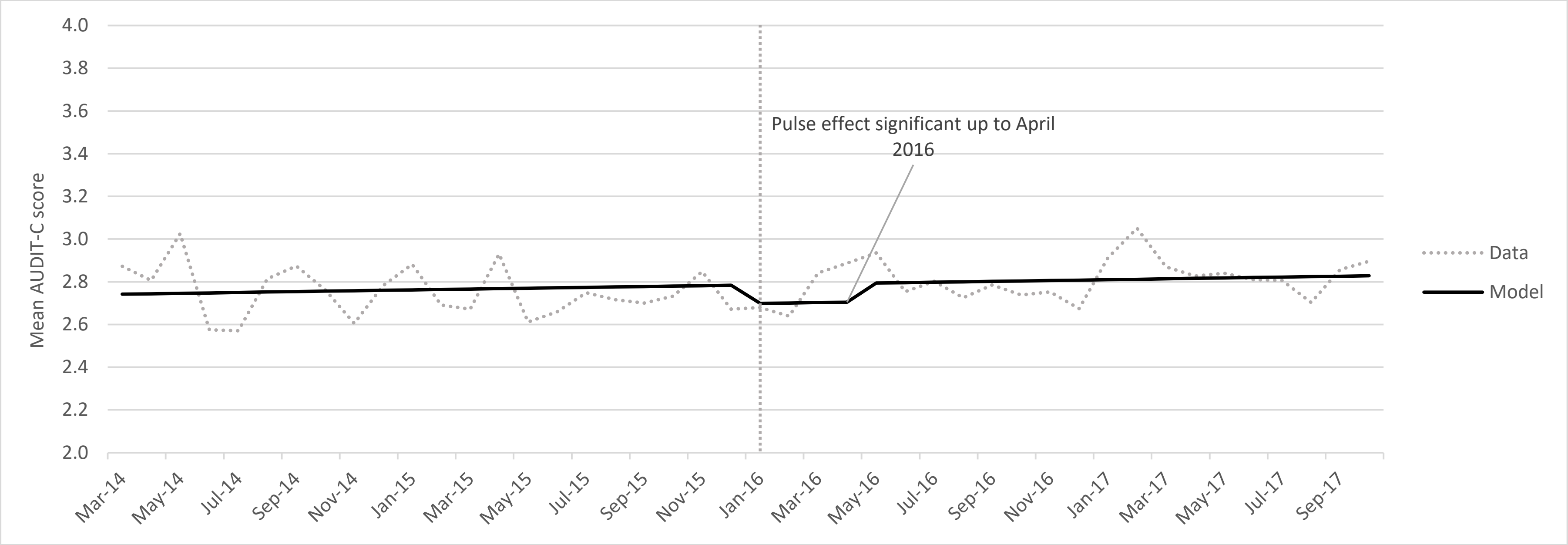

Figure A7: Secondary analysis showing short-term pulse effect on AUDIT-C scores after the announcement of revised drinking guidelines in January 2016.[1]

[1] Model line shows the trend derived from model parameters after controls for seasonality.
